# The Effects of Chitosan-PEG Nanoparticles Based on Channa striata Protein Hydrolyzate on Decreasing Diabetes Mellitus in Diabetic Rats

**DOI:** 10.4314/ejhs.v32i4.21

**Published:** 2022-07

**Authors:** Lintang Dian Saraswati, Bagoes widjanarko, Vivi Endar Herawati, Apriliani Ismi Fauziah

**Affiliations:** 1 Department of Epidemiology, Faculty of Public Health Diponegoro University, Semarang, Indonesia; 2 Department of Health Promotion, Faculty of Public Health Diponegoro University, Semarang, Indonesia; 3 Faculty of Fisheries and Marine Sciences, Diponegoro University, Semarang, Indonesia

**Keywords:** Diabetes mellitus, Chitosan nanoparticles, Channa striata, blood glucose, lipid profile

## Abstract

**Background:**

Channa striata has several good nutrients, including 70% protein, 20% albumin, complete amino acids, zinc, selenium, and iron. However, no study has investigated the chitosan-PEG nanoparticles based on Channa striata protein hydrolyzate. This study's purpose was to determine the role of 10% Channa striata protein hydrolyzate, chitosan-PEG 4000 nanoparticles, and chitosan-PEG 6000 nanoparticles in reducing diabetes mellitus in diabetic rats.

**Method:**

A randomized pretest-posttest control group design was used, with male Sprague-Dawley rats being divided into five groups: STZ, acarbose, hydrolyzate, chitosan-PEG 4000 nanoparticles, and chitosan-PEG 6000 nanoparticles. Diabetes was induced by a single injection of streptozotocin at 1 ml in each formulation. Blood glucose levels were analyzed using a glucometer 7, 14, and 21 days after treatment. The CHOD-PAP method was used to analyze the lipid profile. Pancreas and liver histology analyses were carried out using a microscope.

**Results:**

The formulation of 10% Channa striata protein hydrolyzate and PEG 6000 was the most effective in lowering blood glucose concentrations, and the response was close to the acarbose result. The glucose concentration decreased after daily oral administration of chitosan-PEG nanoparticles for 21 days. The plasma cholesterol, triglycerides, LDL, and HDL concentrations were lower in treated than in untreated diabetic rats.

**Conclusion:**

This study concluded that the formulation of 10% Channa striata protein hydrolyzate and chitosan-PEG 6000 nanoparticles was more effective than acarbose.

## Introduction

Diabetes mellitus is a metabolic disorder characterized by chronic hyperglycemia due to impaired insulin secretion, impaired insulin action, or both (1,2). In type 2 diabetes mellitus, insulin resistance and cell dysfunction are main features (3). Abnormal lipid profile has a close relationship with insulin resistance as indicated by cholesterol, triacylglyceride, high-density lipoprotein (HDL), and low-density lipoprotein (LDL) contents (4,5). Particles at the nanometer scale have distinctive physical properties, especially in improving the drug compounds delivery quality (6). Nanocapsule antidiabetic drugs with nanotechnology have a high bioavailability advantage and the ability to penetrate the intercellular space, which can only be penetrated by petite cell colloid particle sizes (7). The combination of the nanoparticle advantage and various other technologies opens up a considerable potential for development for various purposes and targets. Another advantage is an increase in the system affinity (8).

Chitosan-PEG nanoparticle technology from the protein hydrolyzate of snakehead fish (*Channa striata*) has proven a glucosidase-inhibiting agent (9). Glucosidase inhibitors are oral antidiabetic drugs that help keep blood glucose levels within normal limits, especially after eating (10). In a recent study, we used 10% *Channa striata* protein hydrolyzate, PEG 4000, and PEG 6000 based on *Channa striata* protein hydrolyzate compared with acarbose as a controlled drug, previously circulating and shown to function in controlling blood glucose levels.

## Methods and Materials

**Extract preparation**: Ten percent bromelain enzyme (active pineapple and 85% ethanol) was mixed with 50 grams of snakehead fish meal and 100 ml of distilled water and then incubated in an oven at 55°C for 6 hours (11). Chitosan was made with 1% acetic acid and stirred for approximately 3 hours using the ionic gelation method, then STTP was added. PEG 4000 and PEG 6000 were added to the chitosan nanoparticles in a ratio of 1:3. Hydrolyzate encapsulation was conducted by combining fish hydrolyzate and chitosan nanoparticles at 1:2 (v/v) using the inclusion complexation method.

**Animals and care**: Sprague-Dawley rats (180–250 g) were obtained from the Laboratory of the Animal Center of Gajah Mada University and placed in the Veterinary Laboratory of the Medicine Faculty, Diponegoro University. They were maintained on a standard pellet diet, and the water *ad libitum* in the room was maintained at 25 ± 1 °C.

**Diabetes induction**: Diabetes mellitus in rats was induced by intraperitoneal injection of streptozotocin (STZ) at 50 mg/kg (Sigma, USA) after 8 hours of fasting (12). STZ was dissolved in a citrate buffer (0.01 M, pH 4.5) and stored on ice before use (13). Mice were allowed to drink 5% glucose solution after 6 hours of STZ injection to treat hypoglycemic shock. Five days after STZ administration, mice with fasting blood glucose concentrations of more than 120 mg/dl were considered diabetic and used in the experiment (14).

**Glucose tolerance test**: The rats were divided into five groups (n = 6–9). Group 1 rats were given 1 ml of STZ (50 mg/kg BW) and distilled water. Group 2 mice received 1 ml of STZ (50 mg/kg BW) and acarbose. Group 3 mice received 1 ml of STZ and 10% *Channa striata* protein hydrolyzate. Group 4 mice received 1 ml of STZ and chitosan-PEG 4000 nanoparticles. Group 5 mice received 1 ml of STZchitosan-PEG 6000 nanoparticles (15). After 8 hours, fasting blood glucose levels were determined in the tail blood samples taken on day 0 (before glucose administration), 5 days after induction by intraperitoneal injection with STZ, and 7 days, 14 days, and 21 days after glucose administration (16).

**Plasma cholesterol, triglyceride, HDL, and LDL levels**: Plasma samples were taken on day 21 with the orbital sinus of the eye and collected after centrifugation (3000×g), then stored at -20 °C. Plasma cholesterol, triglyceride, HDL, and LDL levels were measured using Cholesterol Oxidase-Peroxidase Aminoantypirin with absorbance at a wavelength of 546 nm photometer (Shimadzu UV 160A, Japan).

**Taking the pancreases and livers of the rats**: Mice euthanasia was done by putting them in jars given ether so that the mice lost consciousness. Then the rats' neck was broken to prevent the entry of chemicals that would affect the rats' metabolism, and the pancreases were removed. The pancreases taken were then put into organ pots given a 10% BNF solution.

**Pancreatic and liver histopathology procedure**: Liver and pancreatic remains were processed overnight for dehydration, cleansing, and impregnation. Specimens were planted in paraffin blocks, and serial sections 5 m in size were cut using a microtome. The slides were then stained with hematoxylin-eosin and examined under a light microscope at 40x and 100x magnification (Olympus, Japan) (13).

**Statistical analyses**: All data are expressed as means ± standard errors. The data were evaluated by a one-way analysis of variance (ANOVA) using the SPSS program, which was used for mean comparisons, and *P* < 0.05 was considered to be statistically significant.

## Results

**Figure 1:**
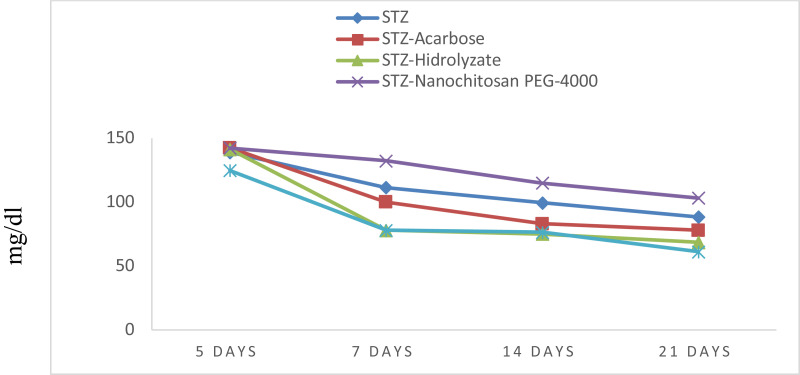
Graph of the average fasting blood glucose levels in all treatment groups

Figure 1 shows that all treatments experienced decreases in glucose levels. The highest reduction rate occurred on day seven after treatment. Although all treatments experienced decreases in glucose levels on days 14 and 21, the decreased values were not higher than day 7.

There were only three treatments with higher glucose-lowering values than streptozotocin: acarbose, hydrolyzate, and chitosan-PEG 6000. Chitosan-PEG 4000 had a much lower glucose reduction value than streptozotocin. Even though together chitosan-PEG 4000 nanoparticles and streptozotocin had a higher glucose-lowering rate than streptozotocin alone, hydrolyzate had a higher ability to lower glucose levels than acarbose and chitosan-PEG 6000 nanoparticles. Table 1 shows a significant difference in the average decrease in blood glucose levels between rats before and after treatment (p-value = 0.000).

**Table 1:** The average fasting blood glucose levels between rat groups before and after treatment

Treatment	Day before and after glucose administration					*P-value*
	
	0	5	7	14	21	
**STZ**	108.89 ± 26.89	139.00 ± 13.15	111.44 ± 7.72	99.44 ± 8.54	88.33 ± 6.50	0.000
**STZ-Acarbose**	115.22 ± 12.85	142.44 ± 25.21	100.00 ± 38.93	83.00 ± 6.29	78.00 ± 7.44	
**STZ-Hydrolyzate**	116.00 ± 6.40	141.33 ± 27.00	78.00 ± 15.13	74.95 ± 17.96	68.56 ± 15.20	
**STZ-Chitosan-** **PEG 4000**	115.11 ± 12.87	142.22 ± 24.21	132.22 ± 14.97	114.89 ± 18.81	103.11 ± 19.77	
**STZ-Chitosan-** **PEG 6000**	122.78 ± 16.20	124.67 ± 60.21	78.00 ± 8.78	76.44 ± 13.48	61.22 ± 14.27	

All treatments experienced decreases in fasting blood glucose levels, in which case the greatest decrease was found in the rats given hydrolyzate and chitosan-PEG 6000 nanoparticles. Meanwhile, at the end of the observation period, it was seen that the fasting blood glucose levels in all groups of rats were below 100 mg/dl, except for the group of rats given chitosan-PEG 4000 nanoparticles.

The rats given chitosan-PEG 6000 nanoparticles and hydrolyzate had the lowest glucose levels on day 21. The rats injected with chitosan-PEG 4000 nanoparticles had the highest fasting glucose levels at the end of the observation period.

The rat group injected with chitosan-PEG 6000 nanoparticles and hydrolyzate had low scores in all tests, except for the triacylglyceride content test. Although the rats given acarbose had lower triacylglyceride contents compared to the rats given all other treatments and control, they showed higher results in cholesterol and low-density lipoprotein levels compared to the control group.

Meanwhile, the chitosan-PEG 4000 nanoparticles treatment had lower values than the values of the control in all tests, but the values remained lower than those of chitosan-PEG 6000 and hydrolyzate. The one-way ANOVA test resulted in a *P*-value of 0.223, which means that there was no significant difference in the average lipid profile between the control and the treatment rat groups.

Figures a1, a2, a3, c1, c2, d3, and d4 show focal necrosis, characterized by losses of tissue structure. Hemorrhagic zones surrounded the necrosis area with bleeding spots. Focal necrosis occurred randomly in single or small cells throughout the liver lobules. Thus, not all lobules were affected (17). The acarbose-treated negative control group showed cell improvement in the islets of Langerhans. Likewise, in the group given chitosan-PEG 4000 nanoparticles based on *Channa striata* protein hydrolyzate, several results show cell improvements in the Langerhans islets (Figures d1 and d2).

**Figure 3:**
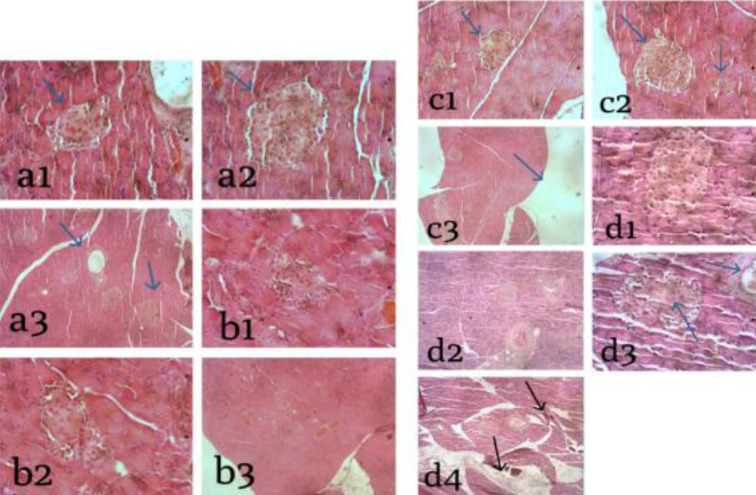
*Histological changes in the pancreas cells* (Notes: a1, a2, a3 = STZ; b1, b2, b3 = Acarbose; c1, c2, c3 = Hydrolyzate; d1, d2, d3, d4 = Chitosan-PEG 4000 nanoparticles).

The livers of mice treated with acarbose were inflamed but not necrotic (Figs. b1,1, and b1,2). Meanwhile, the group of rats given hydrolyzate showed inflammation and necrosis. Inflammation can be seen in Figures c1,3 and c1,4 above, while necrosis can be seen in Figures c1,1 and c1,2, characterized by shrinkage of the cell nucleus so that the cell nucleus looked smaller than its standard size. The necrosis that took place (Fig. c1,1) is called connective necrosis in the pyknosis section. In comparison, (Fig. c1,2) the bridging necrosis in the karyolysis section was characterized by emptying cells due to losses of cell nuclei from the cells. The group of rats treated with chitosan-PEG 4000 nanoparticles showed inflammation and necrosis as well. Inflammation occurred in Figures d1,3 and d1,4, while necrosis occurred in Figures d1,1 and d1,2, characterized by shrinkage of the cell nucleus so that the nucleus looked smaller than its standard size.

**Figure 4:**
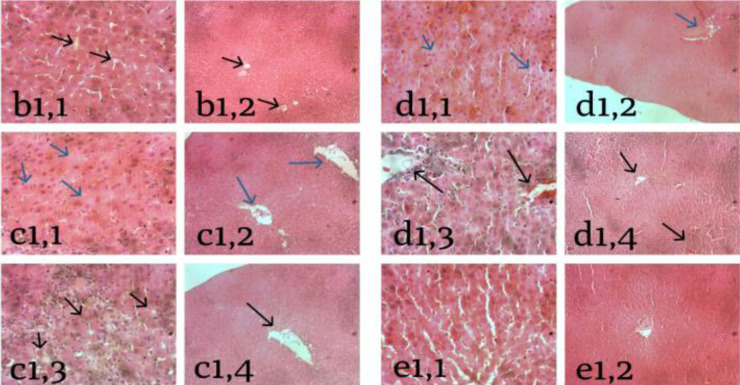
*Histological changes in the liver cells* (Notes: b1,1; b1,2 = Acarbose; c1,1; c1,2; c1,3; c1,4 = Hydrolyzate; d1,1; d1,2; d1,3; d1,4 = Chitosan-PEG 4000 nanoparticles; e1,1; e1,2 = Chitosan-PEG 6000 nanoparticles)

## Discussion

Many studies showed that acarbose lowers postprandial glucose, with decreased fasting blood glucose, plasma triglycerides, and postprandial insulin levels (18,19,20,21). It was found that acarbose could reduce postprandial plasma insulin levels so that acarbose bioavailability is low (22).

Due to the low bioavailability aforementioned, we investigated antidiabetic drugs by nanotechnology in this study. Administration of 10% *Channa striata* protein hydrolyzate was shown to significantly reduce mean blood glucose levels in diabetic rats (Figure 1). These results are similar to the results of previous studies showing that *Channa striata* combined with *Nephelium lappaceum* could reduce blood glucose levels down to 123.3 ± 15.5 mg/dL(23). Another study showed that a combination of *Channa striata* powder and *Zingiber zerumbet* ethanol extract significantly reduced blood glucose levels in diabetic rats (24)

*Channa striata* requires the enzyme bromelain to undertake its hydrolysis process. In this mechanism, the hydrolysis process occurs in a way that the enzyme works specifically and the protein breakdown degree is small. The fermentation process can increase the presence of soluble peptides and amino acids. The inhibition was thought to be due to the role of bioactive compounds in *Channa striata* protein in the form of peptides and amino acids that bind to the enzyme's allosteric (active) side, causing a decrease in the enzyme reaction rate. Therefore, there is a decrease in carbohydrate metabolism into glucose, reducing glucose levels in the blood, which is helpful for people with diabetes mellitus (9).

PEG 4000 and PEG 6000 are characteristic compositions of stabilisers and polymers used for chitosan. In this study, both formulations were used to determine the best formulation that could be used for this antidiabetic drug in lowering blood glucose levels in experimental animals. The encapsulation of chitosan nanoparticles using PEG 4000 and 6000 polymers aims to increase bioavailability. Hydrolyzate, chitosan-PEG 4000 nanoparticles, and chitosan-PEG 6000 nanoparticles could significantly reduce rats' average blood glucose levels (Figure 1, Table 1). Previous studies have shown that insulin-loaded chitosan nanoparticles in specific doses could reduce average blood glucose levels (25,26,27). Oral administration of insulin-loaded chitosanalbumin PEG nanoparticles in diabetic rats causes a decrease in blood glucose levels (28). Nanoparticles increase drug delivery efficiency near the absorption site in the intestine. Mucus penetration particles can improve drug transport and stabilize poorly soluble drugs by encapsulating nanoparticles such as PEG (29). The difference between the present invention and the previous one is the addition of hydrolyzate, chitosan, and PEG to the resulting invention. This invention has gone through several analyses such as FTIR (Fourier Transform InfraRed) analysis, SEM (Scanning Electron Microscopy), and TEM (Transform Electron Microscopy), which showed the stability of the product results.

In conclusion, this study concluded that 10% *Channa striata* protein hydrolyzate formulation and chitosan-PEG 6000 nanoparticles were more effective than acarbose in lowering blood glucose, cholesterol, triglycerides, HDL, and LDL levels and improve the pancreas and liver histology in people with diabetes.

## Figures and Tables

**Figure 2:**
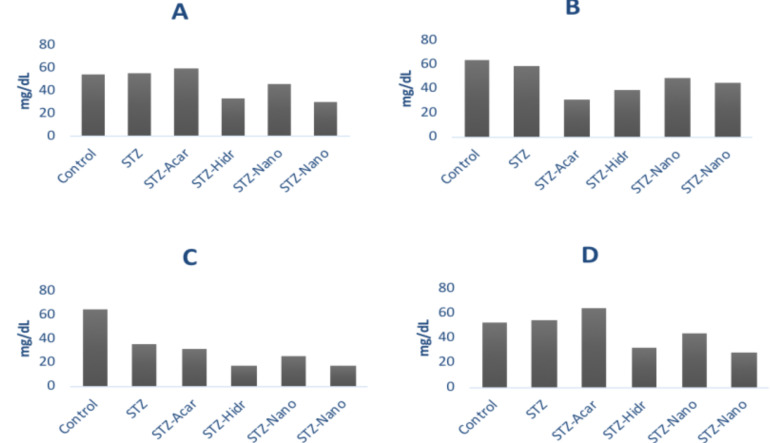
Decreases in average cholesterol (A), triglyceride (B), LDL (C), and HDL (D) levels in all treatment groups.

**Table 2:** The cholesterol, triglyceride, HDL, and LDL level averages in the control and treatment groups

Treatment	Cholesterol (mg/dl)	Triacylglyceride (mg/dl)	LDL (mg/dl)	HDL (mg/dl)	*P-value*
**Control (distilled water)**	53.79 ± 11.61	65.36 ± 22.61	65 ± 17.31	52.26 ± 10.99	0.223
**STZ**	55.83 ± 20.03	59.23 ± 41.70	35.78 ± 17.05	54.09 ± 18.86	
**STZ-Acarbose**	59.82 ± 30.70	30.56 ± 8.22	31.22 ± 19.15	63.92 ± 27.84	
**STZ-Hidrolyzate**	33.25 ± 7.12	39.14 ± 12.88	17.56 ± 7.00	31.81 ± 6.67	
**STZ-Chitosan-PEG 4000**	45.79 ± 11.61	49.10 ± 14.20	25.22 ± 4.84	44.12 ± 11.00	
**STZ-Chitosan-PEG 6000**	29.8 ± 8.97	44.66 ± 3.84	17.78 ± 2.97	28.53 ± 8.25	
